# A Cre‐dependent lentiviral vector for neuron subtype‐specific expression of large proteins

**DOI:** 10.1002/1873-3468.70205

**Published:** 2025-10-28

**Authors:** Weixuan Xue, Sandrine Picaud, Régine Hepp, Stéphanie Pons, Uwe Maskos, Bertrand Lambolez, Ludovic Tricoire

**Affiliations:** ^1^ Sorbonne Université, CNRS, Inserm, Center of Neuroscience Neuro‐SU Paris France; ^2^ Sorbonne Université, CNRS, Inserm, Institut de Biologie Paris‐Seine France; ^3^ Institut Pasteur, Université Paris Cité, Unité Neurobiologie Intégrative des Systèmes Cholinergiques, Centre National de la Recherche Scientifique (CNRS) Paris France

**Keywords:** cell‐specific expression, Cre recombinase, neuron targeting, viral vector

## Abstract

Lentiviral vectors are powerful tools for long‐term expression of large genes in the mammalian brain, but the palette of lentiviral tools available for targeting specific cell subpopulations is restricted. We describe a lentiviral vector for neuronal subtype‐specific expression in Cre mouse lines. Combining a Cre‐dependent flip excision switch with a GFP and a 2A self‐cleaving peptide, it enables identification of living neurons expressing a gene of interest using fluorescence. We validated this vector by targeting neocortical interneuron types and midbrain dopaminergic neurons. Gene expression occurred exclusively in Cre‐expressing neurons without altering their basic electrophysiological properties. This system has been designed to be flexible and easy to modify in order to target expression of any gene of interest in any cell subtype.

## Graphical abstract

We designed a versatile and modular lentivector comprising a Cre‐dependent switch and self‐cleaving 2A peptide and tested it for co‐expression of GFP and a 2.8 kb gene of interest (GOI) in mouse cortical parvalbumin (PV+) interneurons and midbrain dopamine (TH+) neurons. The vector enabled visual identification of targeted neurons in living brain slices and did not alter their electrophysiological properties.

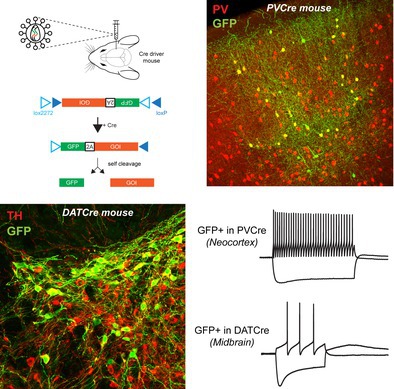

## Abbreviations

2A self‐cleaving peptide

AAV adeno‐associated virus

AHP afterhyperpolarizing potential

DA dopamine

DAT dopamine transporter

FLEX flip excision switch

GFP *Aequorea victoria* green fluorescent protein

GOI gene of interest

IRES internal ribosome entry site

ORF open reading frame

P2A porcine teschovirus‐1 self‐cleaving peptide

PV parvalbumin

SNc substantia nigra pars compacta

SST somatostatin

TH tyrosine hydroxylase

TRPC6 type 6 canonical transient receptor potential channel

VIP vasoactive intestinal peptide

VSV‐G vesicular stomatitis virus glycoprotein

VTA ventral tegmental area

WPRE woodchuck hepatitis virus posttranscriptional response element

WT wild type

During the last two decades, viral vectors for gene transfer have become essential elements in the toolbox of neuroscientists to dissect the complexity of neural networks [1, 2, 3]. In combination with site‐specific recombinases such as Cre recombinase, they have allowed monitoring and manipulating the activity of specific cell populations both in acute slice and *in vivo*. The two main viral tools used so far are recombinant adeno‐associated virus (AAV) and lentivirus. While Cre‐dependent AAV is widely used in neuroscience labs, it suffers from limited cloning capacity (expression cassette < 4.2 kb). This is sufficient to express optogenetic actuators coupled to fluorescent proteins, but it may become quite challenging for the expression of larger inserts such as membrane receptors and ion channels for functional studies. Lentiviral vectors can host larger inserts compared to AAV (expression cassette < 10 kb, [4]) and enable long‐term stable expression of the transgene with minimal deleterious effects on the transduced cells. The larger cloning capacity of lentiviral vectors additionally provides broad opportunities for the use of diverse promoters and regulatory sequences. Despite these advantages, Cre‐dependent lentiviral vectors are much less used than AAV ones.

**Fig. 1:**
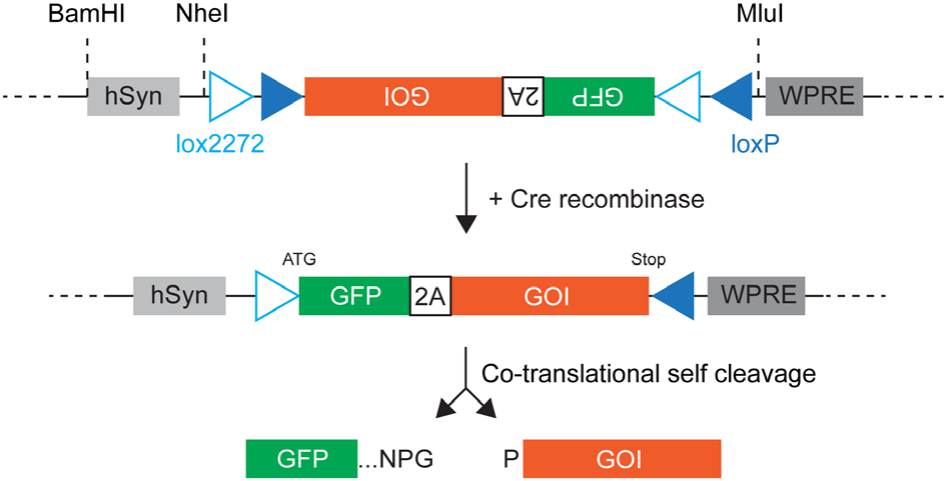
Cre‐dependent lentivector based on a FLEX switch and a self‐cleaving peptide. hsyn: human synapsin I promoter, 2A: porcine teschovirus‐1 self‐cleaving peptide, GOI: gene of interest, WPRE: woodchuck hepatitis virus posttranscriptional response element. GFP and GOI are expressed as a single open reading frame. Note the presence of unique restriction sites separating the promoter and the FLEX expression modules from the lentiviral backbone. *Lower panel*: letters NPG and P stand for amino acids Asparagine‐Proline‐Glycine and Proline that flank the self‐cleavage site of the 2A peptide.

A first series of Cre‐dependent lentiviral vectors relied on a lox‐mCherry‐stop‐lox cassette conferring Cre‐dependent expression of the downstream gene of interest (GOI), which is followed by an internal ribosome entry site (IRES) driving GFP expression [5, 6, 7]. We have used these lentivectors to achieve neuron‐type‐specific, functional expression of neurotransmitter receptor genes, including a 3 kb gene of interest (GOI), which results in a 7.5 kb expression cassette that far exceeds AAV cloning capacity [5, 7, 8]. These vectors nonetheless suffer from drawbacks: (i) a low or variable IRES‐mediated expression of GFP, (ii) the dual mCherry‐GFP expression in Cre‐negative cells that blurs detection of Cre‐positive cells expressing GOI and GFP, but not mCherry. A second series of Cre‐dependent lentiviral vectors uses a different design that circumvents these drawbacks [9, 10, 11]. These vectors are based on a flip excision (FLEX) switch [12], which is routinely used in Cre‐dependent AAV [13] and consists of two pairs of heterotypic, antiparallel loxP‐type recombination sites flanking an inverted open reading frame (ORF). This ORF encodes both the GOI and a fluorescent protein separated by a self‐cleaving peptide [14, 15]. The action of Cre recombinase leads to reversion of the ORF, allowing co‐expression of the two proteins, which are cleaved apart during translation [16]. Recombination also leads to the excision of two recombination sites that leave one of each type of loxP sites oppositely oriented and incapable of further recombination (Fig. 1). These latter lentiviral vectors indeed drive Cre‐dependent co‐expression of GOI and the fluorescent protein, enabling straightforward fluorescence detection. However, these vectors have been tested with GOI of limited size (< 2 kb), and their effects on cellular physiology have not been described [9, 10, 11].

In the present study, we designed a Cre‐dependent lentivector based on a FLEX switch comprising loxP and lox2272 sequences, which shows efficient homotypic but no heterotypic recombination [17], and on the porcine teschovirus‐1 self‐cleaving peptide, which exhibits high cleavage efficiency [18], to achieve consistent co‐expression of GFP and the GOI in specific cell types. The construct also incorporates restriction sites for easy replacement of parts of the expression cassette (promoter, GOI, fluorescent protein) by the end user (Fig. 1). This lentivector was tested for expression of a 2.8 kb GOI encoding a transmembrane ion channel. We show that no GFP leak expression is observed in the absence of Cre recombinase and that the GFP expression level is high enough to allow the detection of GOI‐expressing cells in living and fixed tissues. Furthermore, we provide data regarding the cellular physiology of transduced neurons and the efficiency of the self‐cleaving peptide.

## Materials and methods

### Plasmids and lentiviral vectors

All lentiviral vectors were derived from pLV‐PDGF‐lox‐mCherry‐lox‐EGFP [6] after removal of the PDGF‐lox‐mCherry‐lox GFP cassette by digestion with MluI and BsrGI (Fig. S1) as detailed below. All lentivector plasmids are 2nd generation transfer vectors: the 3'LTR has a deletion in the U3 region (deltaU3) which leads to transcriptional inactivation of the integrated provirus and only mRNA initiated at the internal promoter is expressed. The plasmid pcDNA3.1‐FLEX (Figs S1 and S2), which comprises a FLEX switch made of two pairs of antisense lox sites, one pair of WT loxP and one pair of the mutated lox2272 (Table S1; [13]) was custom made (Genscript) and used to assemble Cre‐dependent expression cassettes before transfer into the pLV‐hSyn‐GFP‐WPRE vector. All PCR primers used in this study are given in Table S1. Plasmid amplification for lentivector production was performed in Stbl3 bacteria (Life Technologies, Villebon sur Yvette, France) grown at 37 °C for up to 12 h. Recombinant lenti pseudo‐virions were produced as described [19] and titers of viral suspensions determined by qPCR on HCT116 cells: 9.4 × 10^8^ infectious genomes (IG)·mL^−1^ (pLV‐hsyn‐GFP), 7.0 × 10^9^ IG·mL^−1^ (pLV‐hsyn‐FLEX‐rev‐GFP), and 2.6 × 10^9^ IG·mL^−1^ (pLV‐hsyn‐FLEX‐rev‐GFP‐2A‐TRPC6).

#### LV‐hSyn‐GFP‐WPRE

hSyn and GFP DNA fragments were obtained by PCR from pLenti‐Synapsin‐hChR2(H134R)‐EYFP‐WPRE (Addgene plasmid #20945; [20]) and pPDGA5 [21], respectively, using Q5 polymerase (NEB) and primers given in Table S1. After purification, PCR products were assembled with lentiviral backbone derived from pLV‐PDGF‐lox‐mCherry‐lox‐EGFP (see above and Fig. S1) using the NEBuilder HiFi DNA Assembly Cloning Kit (NEB). All sequences were verified by DNA sequencing (Fig. S3).

#### LV‐hSyn‐FLEX‐rev‐GFP‐WPRE

GFP was PCR amplified from pPDGA5 [21] and inserted into pcDNA3.1‐FLEX between XbaI and EcoRI sites to give pcDNA3.1‐FLEX‐rev‐GFP. The expression cassette was then excised using NheI and MluI sites and was used to replace GFP in pLV‐hSyn‐GFP‐WPRE at corresponding sites (Figs S1, S4).

#### LV‐hSyn‐FLEX‐rev‐GFP‐P2A‐TRPC6‐WPRE

GFP and TRPC6 sequences were obtained by PCR from pPDGA5 and pcDNA3‐mTRPC6 (generous gift from Mike X Zhu, McGovern Medical School, Texas) and inserted into pcDNA3.1 using NEBuilder HiFi DNA Assembly Cloning Kit to yield pcDNA3.1‐GFP‐P2A‐TRPC6. The GFP‐P2A‐TRPC6 sequence was then excised by digestion with NheI and EcoRI and subcloned into pcDNA3.1‐FLEX between XbaI (compatible with NheI, thereby suppressing both sites) and EcoRI sites to obtain pcDNA3.1‐FLEX‐rev‐GFP‐P2A‐TRPC6. The expression cassette was then excised using NheI and MluI sites and was used to replace GFP in pLV‐hSyn‐GFP‐WPRE at corresponding sites (Fig. S1). The GFP‐P2A‐TRPC6 coding sequence measures 3.6 kb. If we include the hSyn promoter (0.5 kb), WPRE regulatory sequence (0.5 kb), and the FLEX switch (0.3 kb), the size of the whole insert reaches 4.9 kb, which is over the capacity of AAV.

### Lentiviral transduction

Primary culture of mouse cortical neurons was prepared as described [22] and maintained in culture medium (91% High glucose Glutamax supplemented DMEM, 5% FCS, 1% Penicillin/Streptomycin 1000 U·mL^−1^, 1% N2 Supplement, and 2% B27 Supplement (Life Technologies)) for two weeks. Transduction was performed by adding the lentiviral suspension directly into the culture medium. Three days after transduction, cells were fixed with 4% paraformaldehyde (PFA) in 0.12 M sodium phosphate buffer (PB) during 20 min and then washed with Dulbecco's phosphate‐buffered saline (D‐PBS).

For *in vivo* transduction, mice (WT, PV‐Cre [23], SST‐Cre [24] and DAT‐Cre [25]) breeding, handling, and killing were performed in accordance to European Commission guidelines and French legislation (2010/63/UE). The study and all procedures were approved by the French Ministry of Research (Agreement APAFIS#16198‐2018071921137716v3). All efforts were made to minimize the number of animals used during our studies and to ensure their well‐being. Animals were housed in a temperature‐controlled room (21 ± 2 °C, 30–40% humidity) with *ad libitum* access to water and food under a 12 h light/dark cycle. Male and female mice (4–5 weeks) were anesthetized with isoflurane (Iso‐Vet, Piramal Healthcare UK) and restrained in a stereotaxic apparatus. Mice were injected with lentiviral suspension (1 μL per site) at following coordinates in mm from bregma (AP), skull surface (DV), and midline (ML). For hippocampus: −1.8 AP, −1.5 DV, 1.35 ML; for neocortex: 1.4 AP, 0.6 DV, 2.4 ML; for ventral tegmental area (VTA) and substantia nigra pars compacta (SNc): −3 to −3.1 AP, −4.5 to −4.6 DV, 0.5 ML.

### Whole cell electrophysiological recordings

One month after injection, mice were deeply anesthetized with an intraperitoneal injection of euthasol (150 mg·kg^−1^ body weight), and 120 U of heparin (Sigma, Saint‐Quentin‐Fallavier, France) was injected into the left heart to prevent coagulation. Then, mice were intracardiacally perfused with cold (4 °C) sucrose artificial cerebrospinal fluid (ACSF) containing (in mm): 30 sucrose, 2.5 glucose, 126 NaCl, 2.5 KCl, 26 NaHCO_3_, 3 NaH2PO_4_, 3 MgCl_2_, 0.5 CaCl_2_, and 3 kynurenic acid. Brains were quickly removed, and slices (250 μm thick) were prepared in sucrose ACSF using a vibratome (VT1000S, Leica, Nanterre, France). Slices were transferred to a chamber containing standard ACSF, saturated with 95% O_2_ and 5% CO_2_, and containing (in mm): 126 NaCl, 2.5 KCl, 26 NaHCO_3_, 3 NaH2PO_4_, 1 MgCl_2_, 2 CaCl_2_, 10 sucrose, and 10 glucose. Slices were allowed to recover for 30 min at 34 °C and then incubated at room temperature (20–25 °C) until recording. Patch pipettes (3–5 MΩ) were pulled from borosilicate glass (World Precision Instruments) on a micropipette puller (Model PP‐83; Narishige, London, UK). Recordings were performed at room temperature with pipettes filled with internal solution containing (in mm): 144 KGlu, 3 MgCl_2_, 10 HEPES, 0.5 EGTA, and 2 mg·mL^−1^ biocytin. The pH was adjusted to 7.2, and the osmolarity to 295 mOsm. Transduced neurons were selected under epifluorescence illumination with a 470 nm LED (CoolLED, Andover, UK) and a GFP filter set (Semrock, West Henrietta, NY, USA). Whole cell patch‐clamp recordings were made on cells visualized under infrared videomicroscopy with Dodt contrast, using a Multiclamp 700B amplifier and Digidata 1440A analog‐to‐digital converter (Molecular Devices, San Jose, CA, USA). Signals were filtered at 1–5 kHz, digitized at 20 kHz, saved to a personal computer, and analyzed off‐line with clampfit 10.2 software (Molecular Devices).

To probe the presence of H‐current, cells were held at −50 mV and subjected to a 1 s long hyperpolarizing voltage step (from −100 mV to −50 mV by 10 mV increments). To assess firing properties, cells were recorded in current clamp mode and subjected to 800 ms‐long current steps (ranging from −100 pA to +200 pA in 25 pA increments in DAT‐Cre mice, and from −200 pA to +500 pA in 50 pA increments in PV‐Cre mice). Action potential amplitude was defined as the difference in membrane potential between the threshold and the peak. Afterhyperpolarization (AHP) amplitude was defined as the difference between the action potential threshold and the most negative membrane potential attained during the AHP. These properties were obtained from the first action potential elicited by a depolarizing 800 ms‐long current pulse of just suprathreshold strength. Firing frequency was calculated based on the response evoked by a current pulse of twice the threshold strength, as the inverse of the mean of the last two to three interspike intervals. Membrane resistance was measured by applying a depolarizing current pulse (amplitude 50 pA, duration 800 ms). The time constant was determined by fitting voltage responses to the same pulse with a single exponential function. Membrane potentials were not corrected for junction potential.

At the end of the recording, slices were fixed with 4% paraformaldehyde (PFA) in 0.12 M sodium phosphate buffer (PB) overnight at 4 °C and then transferred to D‐PBS.

### Immunohistochemistry

Primary antibodies: chicken anti‐GFP (1 : 1000, Cat # GFP‐1010; Aves Labs, Davis, CA, USA), mouse anti‐TH (1 : 1000, clone LNC1, Cat # MAB318; Merck Millipore, Darmstadt, Germany), mouse anti‐microtubule associated protein 2 (MAP2, 1 : 500, clone AP20, Cat # MAB3418; Merck Millipore), rabbit anti‐PV (1 : 1000; Swant, Kehrsatz, Switzerland), goat anti‐SST (1 : 1000, Cat # D‐20; Santa Cruz, Dallas, Texas, USA).

Primary neurons: cells were permeabilized during 30 min in D‐PBS/0.2% Gelatin/0.3% TritonX‐100 (PBS‐GT) and incubated for 1 h with primary antibodies diluted in PBS‐GT. After several washes in PBS‐GT, cells were incubated for 1 h with secondary antibodies diluted in PBS‐GT. Nuclear counterstaining was performed by treating cells for 20 min with 4′,6‐diamidino‐2‐phenylindole (DAPI, 100 ng·mL^−1^) diluted in PBS. Cells were washed several times in PBS and mounted in Fluoromount‐G (Clinisciences, Nanterre, France).

For biocytin staining of recorded neurons, slices were processed as described [8].

Immunohistochemistry for fluorescence microscopy: mice were deeply anesthetized with intraperitoneal injection of euthasol (150 mg·kg^−1^ body weight) and intracardiacally perfused with 4% paraformaldehyde/0.12 M phosphate buffer pH 7.6. Brains were postfixed for 2 h in the same fixative and rinsed in D‐PBS. Brains were sliced to 50 μm thickness using a vibratome (VT1000S; Leica microsystem) and kept in D‐PBS at 4 °C. Free‐floating sections were blocked for 2 h at room temperature in a PBS/0.25% Triton X‐100/0.2% gelatine solution (PBS‐GT) before being incubated overnight (extended to 48 h for SST immunostaining, [26]) at 4 °C with primary antibodies diluted in PBS‐GT. Slices were washed with PBS‐GT before being incubated for 2 h at room temperature with secondary antibodies diluted in PBS‐GT. After extensive washing in PBS, slices were mounted in Fluoromount‐G (Clinisciences, France). Secondary antibodies: goat anti‐chicken IgG alexafluor488 (1 : 1000; Life Technology), goat anti‐mouse IgG rhodamine Red‐X (1 : 1000; Jackson Immunoresearch, Ely, UK), goat anti‐rabbit IgG rhodamine Red‐X (1 : 000; Jackson Immunoresearch) and donkey anti‐goat IgG Cy5 (1 : 500; Jackson Immunoresearch).

Images were acquired using a confocal laser scanning microscope (SP5, Leica), and processed with the imagej software.

### Western blot

Calcium phosphate‐transfected HEK293T cells (ATCC Number: CRL‐3216, RRID:CVCL_0063, authenticated in the past three years using Short Tandem Repeat analysis by the ATCC cell authentication service) were lysed in a buffer containing 50 mm Tris/HCl pH 7.5, 150 mm NaCl, 1% Nonidet P40, 0.5% Sodium deoxycholate, and protease inhibitors for 1 h. All experiments were performed with mycoplasma‐free cells. Protein concentration was determined with Bradford's method using BSA as standard. Equal protein amounts (10 μg per lane) were separated by SDS/PAGE on 4–15% polyacrylamide gels (Biorad, Hercules, CA, USA) and transferred onto nitrocellulose sheets. Protein loading was controlled by reversible Ponceau Red staining. Nonspecific sites on nitrocellulose membranes were blocked for 1 h at room temperature with PBS containing Tween‐20 (0.1%) and 5% BSA. Membranes were incubated overnight with primary antibodies at 4 °C. The primary antibodies used were rabbit anti‐TRPC6 (Alomone Labs, Jerusalem, Israel, ACC‐017, 1/1000), rabbit anti‐GFP (Chromotek, Martinsried, Germany, PABG1, 1/1000), and mouse anti‐Actin (Sigma, A1978, 1/10000). Bound antibodies were detected with secondary Goat anti‐IgG antibodies coupled to either DyLight800 (Thermofisher scientific, Waltham, MA, USA, SA5‐10036, 1/2000) or DyLight680 (#35519, 1/2000) and analyzed with the LI‐COR Odyssey detection system.

## Results

Starting from the plasmid pLV‐PDGF‐lox‐mCherry‐lox‐EGFP [7], we aimed at generating a user‐friendly and flexible lentiviral vector for robust, neuron‐specific, Cre‐dependent expression. The expression cassette was first removed, leaving only the HIV‐derived backbone and the WPRE sequence (see Methods). Using seamless DNA assembly, the human synapsin I promoter [27] and a GFP coding sequence were then inserted upstream of the WPRE to obtain the plasmid pLV‐hSyn‐GFP‐WPRE (see Methods, Figs S1 and S3). During this process, unique NheI and MluI sites were created at each end of the GFP sequence, respectively. Notably, a unique BamHI site is located just upstream of the hSyn promoter in pLV‐hSyn‐GFP‐WPRE. This construct thus exhibits three unique restriction sites that divide the expression cassette into two independent modules, promoter and coding sequence, which can be exchanged with other elements using simple cloning steps (Figs S1 and S3). To validate this construct, we transduced primary cultures of mouse cortical neurons with lentivirions derived from pLV‐hSyn‐GFP‐WPRE. Since cultures used in this study contain both neurons and glial cells, we performed immunostaining against the neuron‐specific marker MAP2 [28]. All GFP‐positive cells were also MAP2‐positive (*n* = 35), confirming neuron‐specific expression of the lentiviral transgene (Fig. S5A). To validate this construct further, the lentiviral vector was injected into the hippocampus of one‐month‐old mice. Immunohistological analyses revealed intense GFP expression in CA1 pyramidal neurons. In the dentate gyrus, granule cells were labeled, as well as a few cells in the molecular layer, likely GABAergic interneurons (Fig. S5B). Apical dendrites of CA1 pyramidal cells in stratum radiatum and lacunosum moleculare were clearly visible (Fig. S5B). We did not observe GFP‐expressing cells exhibiting glial cell morphology. These observations indicate that the LV‐hSyn‐GFP‐WPRE lentivector efficiently drives transgene expression in neurons, in agreement with previous reports using the hSyn promoter [27, 29].

**Fig. 2:**
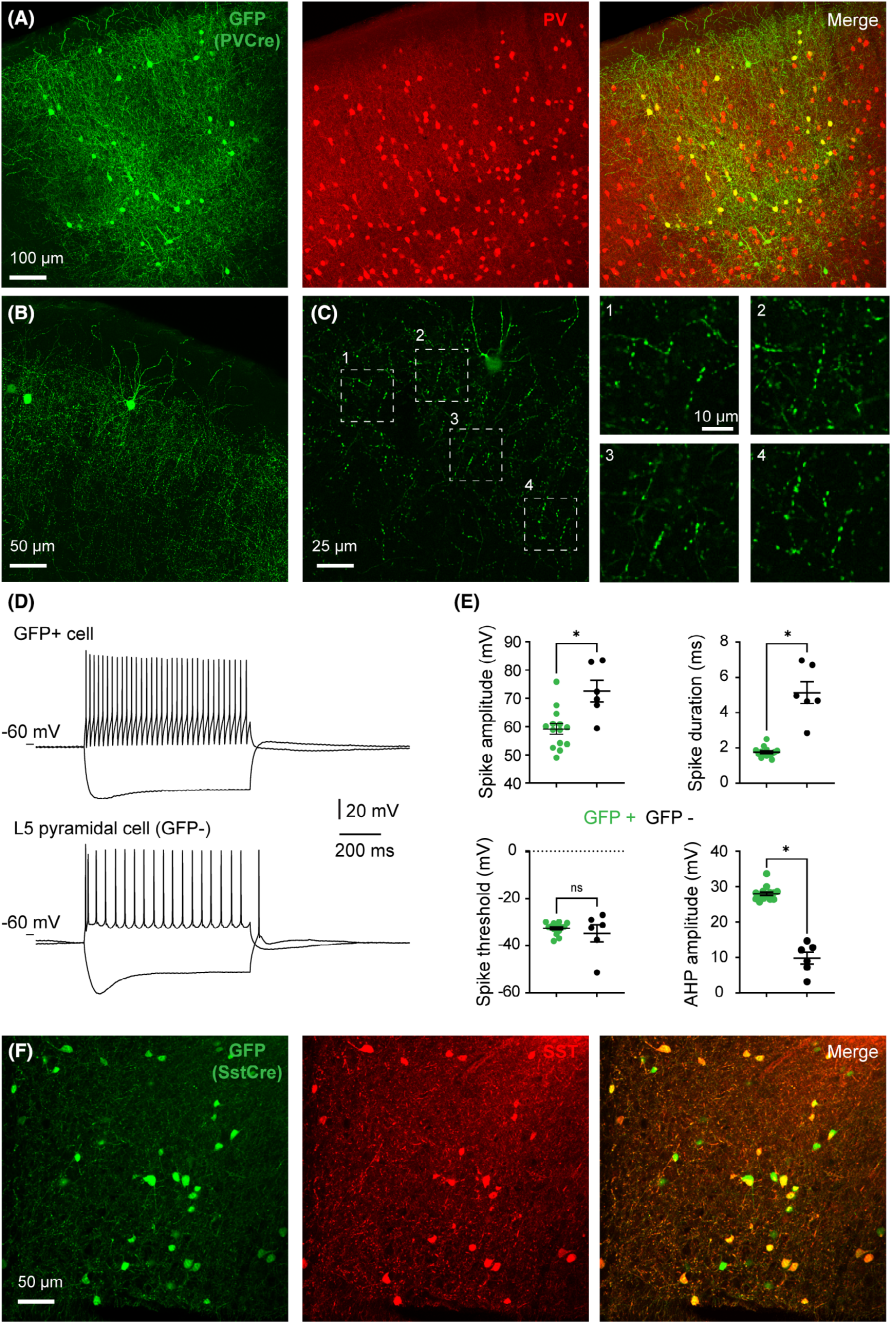
Validation of LV‐hSyn‐FLEX‐rev‐GFP lentivector in the neocortex of Cre driver mice. Results obtained in PV‐Cre (panels A–E) and SST‐Cre (panel F) mice. (A) Z‐projection of a confocal stack showing expression of GFP and PV in the neocortex one month after injection of lentiviral particles. (B) Z‐projection of a confocal stack illustrating GFP+ neurons with clearly visible axonal arborization. (C) Single frames from the confocal stack in B illustrating the labeling of axon terminals. (D) Firing pattern of layer 5 GFP+ cells and neighboring pyramidal cells in response to current injection (−200 pA; +200 pA). (E) Comparison of electrophysiological parameters. Note the brief spikes and large AHPs of GFP+ cells (*n* = 13 GFP+ cells, *n* = 6 pyramidal cells). Error bars represent standard error of the mean. Asterisk indicates *p*‐value < 0.05 (*t*‐test). (F) Z‐projection of a confocal stack showing expression of GFP and SST in the neocortex one month after injection of lentiviral particles.

Next, we inserted a Cre‐dependent GFP expression cassette in the lentivector to generate pLV‐hSyn‐FLEX‐rev‐GFP‐WPRE (see Methods, Fig. S1 and Fig. S5). To validate this latter vector, we injected corresponding lentivirions in the neocortex of PV‐Cre mice, which express Cre recombinase in cortical parvalbumin (PV)‐expressing, GABAergic interneurons [23]. One month after lentiviral injection, the specificity of GFP expression in PV‐expressing neurons was assessed using dual immunolabeling. We observed preferential expression of GFP in PV neurons in all cortical layers, with 84% of GFP‐positive cells also being positive for PV (*n* = 610, Fig. 2A). A large fraction of GFP‐positive cells exhibited non‐pyramidal somatodendritic morphology, as expected from GABAergic interneurons (Fig. 2A,B). However, some GFP+ cells exhibiting pyramidal‐like morphology with a prominent apical dendrite could also be observed (see Fig. 2A). This is presumably due to the low expression of PV in pyramidal cells [30, 31], which is also reported in several other types of GABAergic interneurons [32, 33, 34]. GFP extensively labeled the dendrites of transduced neurons and was also present in presumptive axons and axon terminals (Fig. 2B,C).

We next prepared acute neocortical slices from PV‐Cre mice injected with LV‐hSyn‐FLEX‐rev‐GFP‐WPRE lentivirions and found that the fluorescence was strong enough to allow visual identification of living, GFP‐positive neurons using a regular GFP filter set. Using whole‐cell patch‐clamp recording, we measured basic active and passive electrophysiological properties of layer 5 GFP‐positive cells and compared them to those of GFP‐negative pyramidal cells of the same layer (Fig. 2D). In agreement with previous characterization of PV interneurons [33], GFP cells exhibited action potentials of smaller amplitude and duration with characteristically larger afterhyperpolarization compared to pyramidal cells (*n* = 13 GFP+ cells, *n* = 6 pyramidal cells, Fig. 2E). These data indicate that the LV‐hSyn‐FLEX vector is suitable to target PV‐expressing interneurons in the neocortex.

To evaluate whether this lentivector is able to transduce other types of GABAergic interneurons, we injected the LV‐hSyn‐FLEX‐rev‐GFP vector in the neocortex of SST‐Cre mice, which express Cre recombinase in somatostatin (SST)‐expressing GABAergic interneurons [24]. One month after lentiviral injection, the specificity of GFP expression in SST‐expressing neurons was assessed using dual immunolabeling. We observed preferential expression of GFP in SST neurons in all cortical layers, with 68% of GFP‐positive cells being also positive for SST (*n* = 884, Fig. 2F). Both SST‐positive and negative GFP‐expressing cells essentially exhibited non‐pyramidal somatodendritic morphology, as expected from GABAergic interneurons (Fig. 2F). The non‐immunodetection of SST in a fraction of GFP+ cells is likely due to the downregulation of SST expression in the SST‐Cre mouse line [26] and to the weak expression of SST in a subset of PV interneurons [32, 33, 35]. These data indicate that the present LV‐hSyn‐FLEX vector effectively targets specific neuron types in the neocortex using suitable Cre driver mouse lines.

**Fig. 3:**
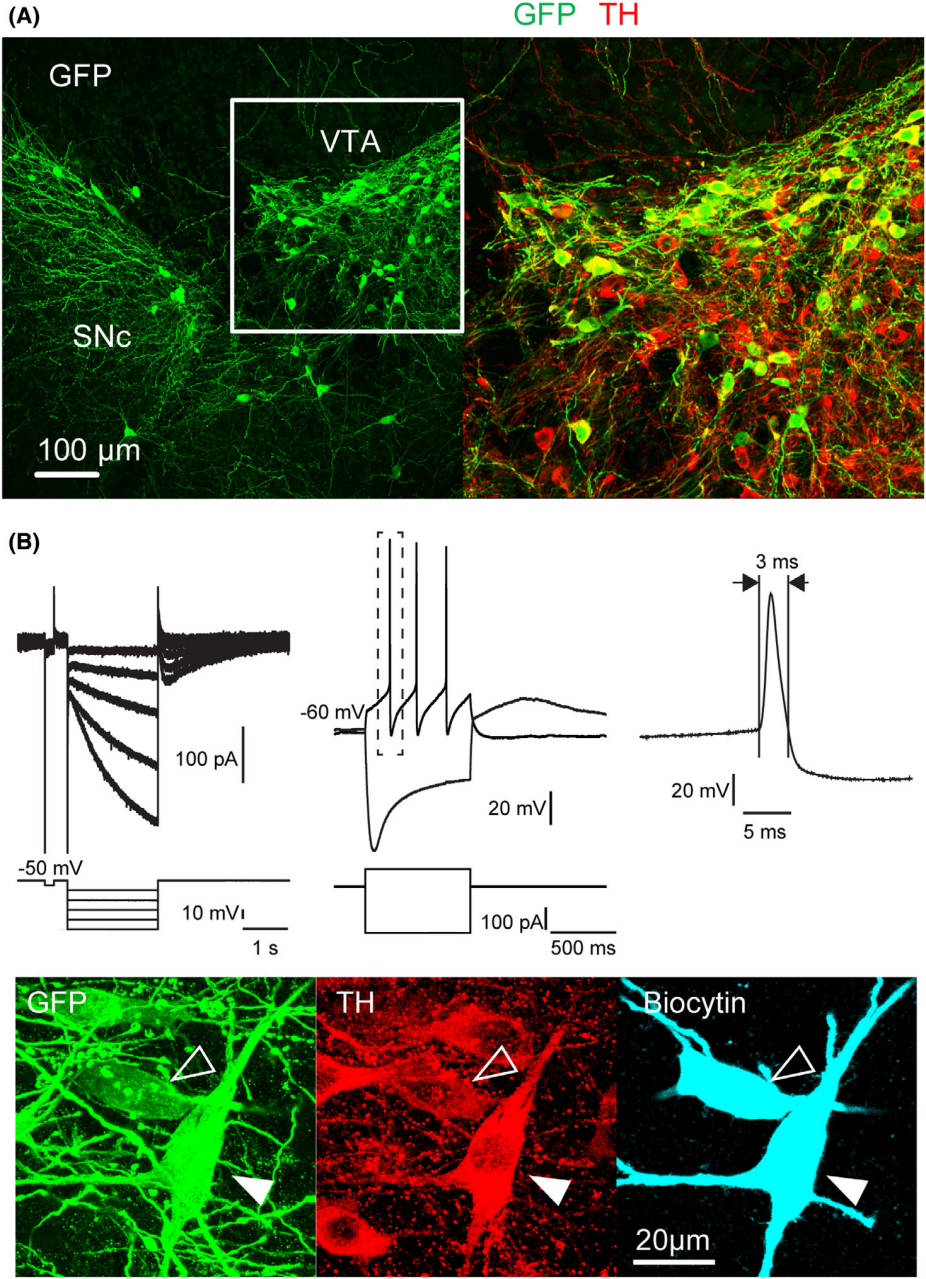
LV‐hSyn‐FLEX‐revGFP‐WPRE targets dopaminergic (DA) neurons of DAT‐Cre mice. (A) Confocal stack illustrating the expression of tyrosine hydroxylase (TH, red) and GFP (green) in the ventral tegmental area and substantia nigra pars compacta (VTA and SNc) of DAT‐Cre mice one month after local injection of lentiviral particles. (B) Upper: electrophysiological characterization of a GFP‐positive neuron from the SNc exhibiting large Ih current (left) and long duration action potentials typical of DA neurons. Lower: upon histochemical examination, this recorded and biocytin‐filled GFP‐positive neuron (filled arrowhead) was TH immunopositive, attesting to its DA phenotype. Note the presence of another recorded and biocytin‐filled DA neuron (empty arrowhead) with lighter GFP and TH staining, possibly due to cytosolic washout during longer recording.

We further validated the LV‐hSyn‐FLEX‐rev‐GFP‐WPRE vector by injecting the lentiviral suspension in the VTA of DAT‐Cre mice [25]. This mouse line expresses Cre recombinase under the control of the dopamine transporter promoter, which drives Cre expression specifically in dopaminergic (DA) neurons [25]. One month after lentiviral injection, we examined the specificity of GFP expression in DA neurons using dual immunolabeling of GFP and the dopamine synthesizing enzyme, tyrosine hydroxylase (TH). All GFP immunopositive neurons were also TH immunopositive (*n* = 52, Fig. 3A). We also prepared acute midbrain slices and examined the intrinsic electrophysiological properties of GFP‐positive cells in the VTA (*n* = 6) using whole cell patch‐clamp recording. As expected for DA neurons, cells exhibited a large inward H‐current upon transient hyperpolarization in voltage clamp mode (Fig. 3B). In current clamp mode, negative step current injection evoked a hyperpolarization exhibiting a prominent sag consistent with the activation of H‐current. Suprathreshold depolarization induced by positive current injection elicited low frequency firing of long duration action potentials (2–3 ms, Fig. 3B). These properties are consistent with the typical phenotype of DA neurons [36, 37, 38]. The identity of recorded DA neurons was confirmed by *post hoc* TH immunostaining and biocytin revelation (*n* = 6, Fig. 3B). These results demonstrate specific, Cre‐dependent GFP expression with our FLEX lentivirus, which allowed identification of virally transduced living neurons in acute slices and did not conspicuously alter the basic electrophysiological properties of cortical PV interneurons and midbrain DA neurons.

**Fig. 4:**
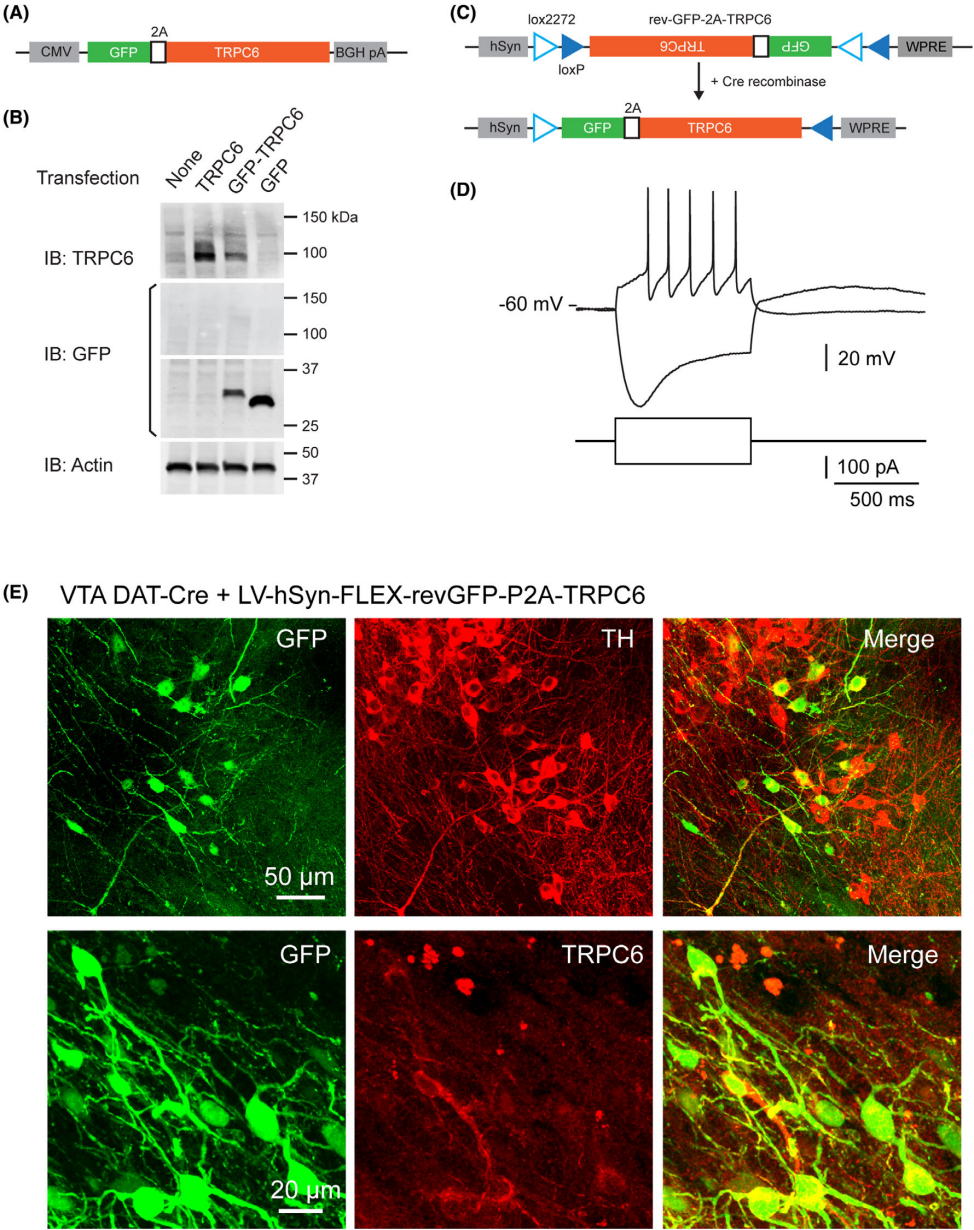
LV‐hSyn‐FLEX‐rev‐GFP‐P2A‐TRPC6‐WPRE allows GFP and TRPC6 expression in dopaminergic (DA) neurons of DAT‐Cre mice. (A) GFP‐P2A‐TRPC6 cassette in mammalian expression plasmid pcDNA3.1. (B) Western blot analysis of protein lysates from HEK cells transfected with plasmids expressing GFP‐P2A‐TRPC6, GFP alone, or TRPC6 alone. Note the absence of GFP labeling in the 100–150 kDa range corresponding to TRPC6 and the higher molecular weight of the GFP band in the GFP‐TRPC6 lane. (C) GFP‐P2A‐TRPC6 cassette in the FLEX lentivector. (D) Trace illustrating electrophysiological characterization of GFP+ neurons in the ventral tegmental area (VTA) of DAT‐Cre mice one month after local injection of lentiviral particles. (E) Confocal stack illustrating the co‐expression of GFP and tyrosine hydroxylase (TH), and of GFP and TRPC6 in VTA neurons.

We next examined the usefulness of our lentiviral vector for cell‐type‐specific expression of large proteins. We generated a single ORF consisting of GFP and type 6 canonical transient receptor potential channel (TRPC6) sequences separated by the self‐cleaving 2A peptide sequence (Fig. 4A, length of the coding sequence: 3573 bp). During translation, self‐cleavage of the polypeptide between glycine and the second proline in the NPGP motif of the 2A sequence [16] results, in principle, in equimolar amounts of GFP harboring an 18 amino acid tail that does not alter its fluorescence [18], and of TRPC6 harboring an additional proline at its N terminus.

We first cloned the GFP‐2A‐TRPC6 sequence in the pcDNA3.1 plasmid and transfected this construct in 293 HEK cells, in parallel with control plasmids containing either GFP or TRPC6 alone. Analyses of GFP and TRPC6 expression in 293 HEK cell lysates were performed by western blot. The GFP band from the GFP‐2A‐TRPC6 lysate exhibited a slightly higher molecular weight than the band from the GFP‐alone lysate, consistent with the addition of the 2A tail after self‐cleavage. Extensive self‐cleavage of the GFP‐2A‐TRPC6 polypeptide was assessed by the absence of a GFP band in the 100–150 kDa range corresponding to TRPC6 (Fig. 4B). Likewise, the TRPC6 band from the GFP‐2A‐TRPC6 lysate had the same molecular weight as the TRPC6 band from the TRPC6‐alone lysate (Fig. 4B). These results indicate that GFP and TRPC6 are efficiently expressed and cleaved apart from the GFP‐2A‐TRPC6 sequence.

The GFP‐2A‐TRPC6 sequence was then used to generate pLV‐hSyn‐FLEX‐rev‐GFP‐2A‐TRPC6 (see Methods, Figs 4C and S1), and the corresponding lentivirions were injected in the VTA/SNc of DAT‐Cre mice (see Methods). One month after injection, the GFP fluorescence was clearly visible in living midbrain slices. Using whole cell recordings, we found that GFP+ neurons (*n* = 6) exhibited a large sag indicative of H‐current and relatively long action potentials (Fig. 4D) typically observed in DA neurons [36, 37, 38]. Immunostaining against GFP and the DA synthesizing enzyme, tyrosine hydroxylase (TH), indicated that all GFP‐positive neurons coexpressed TH (*n* = 23, Fig. 4E), consistent with selective transgene expression in DA neurons. GFP‐positive neurons also expressed TRPC6 (*n* = 15, Fig. 4E), confirming correct recombination and expression of the entire ORF. These data indicate that the present Lenti FLEX vector enables Cre‐dependent expression of large GOI such as ion channels together with a fluorescent reporter with high cell type specificity and minimal alteration of electrophysiological properties of targeted cells.

## Discussion

We have developed a lentiviral vector that allows cell‐type‐specific expression of large proteins together with GFP by combining a Cre‐dependent approach with a 2A self‐cleaving peptide. It is based on a modular and versatile construct, in which the GOI, the promoter, or the fluorescent reporter protein can be easily replaced. The lentiviral vector enabled straightforward identification of targeted neurons in living brain slices and did not conspicuously alter the electrophysiological properties of PV‐expressing or DA neurons. This vector is a useful tool for the cell‐type‐specific control of molecular mechanisms and the study of their role in brain circuits.

Over the past decades, the transduction efficiency, tropism, and versatility of lentiviral vectors have been substantially improved. For example, pseudotyping (i.e., replacing the glycoprotein of the viral envelope by that derived from other viruses) with the vesicular stomatitis virus glycoprotein (VSV‐G) has resulted in wide tropism and in the ability to transduce both glia and neurons [39]. Efforts to restrict lentiviral transduction to cell subtypes initially involved the use of mini‐promoters. Although successful in some cases (CaMKIIa, ORX, GAD [40, 41, 42]), this approach is limited by mini‐promoters' availability and sometimes results in off‐target or inconsistent expression [43]. The development of multiple Cre driver mouse lines has set the gold standard for genetic targeting of specific cell types throughout the brain [24]. These tools have been extensively exploited for cell‐type‐specific gene transfer with Cre‐dependent AAV, but far less with Cre‐dependent lentivirus [5, 6, 7, 8, 9, 10, 11]. In the present study, we have tested our Cre‐dependent lentivector in three neuron types specifically targeted using PV‐Cre, SST‐Cre, and DAT‐Cre mice. We found that the lentivector drives robust GFP expression essentially restricted to PV or SST GABAergic interneurons, as expected from the use of the corresponding Cre mouse line [23, 24]. This was assessed by the morphology of GFP‐expressing neurons and their PV or SST immunopositivity and, in the case of PV interneurons, by action potential firing properties, which were similar to those described [33]. This observation further indicates that lentiviral transduction does not conspicuously alter the basic properties of PV interneurons. GABAergic interneurons represent ~20% of cortical neurons and play a critical role in gating afferent inputs and synchronizing the activity of principal excitatory neurons [44, 45]. Cre‐dependent AAV has been extensively used to probe the role of interneurons in cortical circuits using opto‐ or chemogenetic tools. Owing to its larger cloning capacity, Cre‐dependent lentivectors thus represent a useful complement to AAV, enabling analysis of the role played by various molecules in the function of interneurons and their contribution to cortical circuits. A previous report indicates that VSV‐G pseudotyped lentiviral vectors less efficiently transduce GABAergic interneurons than excitatory neurons in the neocortex [43]. Although our results do not allow comparing transduction efficiencies between neuron types, we clearly show, using moderate virion titers (see Methods), that our lentivector enables efficient gene transfer in PV and SST interneuron types, which account for ~40% and ~30% of neocortical interneurons, respectively [44]. An earlier version of the Cre‐dependent lentivector based on a lox‐mCherry‐stop‐lox cassette followed by an IRES driving GFP expression has been used to express transgenes specifically in DA neurons in DAT‐Cre mice [5, 7, 8]. The present results show that our FLEX‐based lentivector resulted in robust GFP expression selectively in targeted DA neurons and confirm that lentiviral transduction does not conspicuously alter their electrophysiological properties.

The 2A self‐cleaving peptides are 18–22 amino acid sequences that are now widely preferred to IRES, which sometimes results in low or variable efficiency for the expression of two or more proteins from a single ORF. These 2A peptides produce separate proteins at equimolar amounts from a single ORF by inducing co‐translational “ribosomal skipping” [16, 46, 47, 48, 49]. Several 2A peptides have been isolated from different viruses, and it is possible to express more than two proteins with a single ORF by using multiple 2A sequences, even if the proteins are targeted to different subcellular compartments [18, 50]. This 2A peptide approach has been previously used to co‐express a GOI and a fluorescent protein upon lentiviral transduction in diverse cell types, with the 2A peptide being placed either upstream or downstream of the GOI [9, 10, 11, 51, 52]. In the present lentivector, the porcine teschovirus 2A peptide follows GFP and is upstream of TRPC6. In this configuration, self‐cleavage of the 2A peptide, which occurs near its C‐terminus [18], adds only a proline to the native N terminus of the GOI, which is unlikely to hamper the proper subcellular targeting and functional properties of the GOI. Indeed, this design has allowed functional expression of a receptor‐channel comprising an N‐terminal signal peptide through lentiviral transfer, which restored the normal phenotype in a mouse model of cerebellar ataxia [51]. Here, we found that expression of the GFP‐2A‐TRPC6 sequence in HEK cells resulted in extensive self‐cleavage, with no detection of the uncleaved sequence upon western blot analysis. We also found that GFP exhibited an increased molecular weight, consistent with the addition of an 18 amino acid tail resulting from 2A peptide self‐cleavage at the expected position. Gene transfer of the GFP‐2A‐TRPC6 sequence in midbrain neurons of DAT‐Cre mice using our FLEX‐based lentivector resulted in co‐expression of GFP and TRPC6 in transduced neurons. Transgene expression was selective for DA neurons, and GFP expression allowed straightforward identification of transduced DA neurons for electrophysiological analysis in living midbrain slices, which assessed the good viability of these neurons.

Owing to its relatively large cloning capacity and to the presence of unique restriction sites, the present lentivector is flexible and modular. The ORF can accommodate larger GOI than TRPC6, the 2A peptide allows a variety of subcellular targeting, and GFP can be replaced by a fluorescent intracellular sensor or complemented by any optical sensor pending the addition of a second 2A peptide. The present lentivector can also be transformed into a “Cre‐OFF” vector, for which expression is suppressed by Cre activity [53], by inserting the ORF sequence in the forward orientation in the FLEX switch. Furthermore, Cre‐dependent loxP sites can be combined with FLP‐dependent FRT recombination sites, so as to achieve intersectional targeting of cell populations defined by two molecular criteria [54, 55]. Finally, in order to target nonneuronal cell types, the hSyn promoter of our lentivector can be replaced by any desired promoter, such as ubiquitous promoters of elongation factor 1 alpha (EF1α) or phosphoglycerate kinase (PGK) genes, as long as the expression cassette does not exceed 10 kb [4]. It is noteworthy that an important advantage of lentivirus over AAV is its ability to integrate into the genome of both dividing and non‐dividing cells. Hence, the present Cre‐dependent lentivector offers more flexibility than corresponding AAV in terms of cloning capacity and the variety of targetable cell types. As compared to lentivirus, however, AAV diffuses more broadly upon injection in living tissue due to its smaller size and, in the current state of the art, benefits from a larger choice of pseudotypes to optimize the transduction of targeted cell types [56, 57].

In conclusion, our Cre‐dependent lentivector showed excellent specificity with no leakiness in off‐target cells and efficient co‐expression of GFP and TRPC6. This lentivector can be easily modified to target the expression of different genes in various cell types. The present lentivector can be used by a broad scientific community as a versatile tool for the cell‐type‐specific study of molecular mechanisms and their functional impact *in vivo*.

## Conflict of interest

The authors declare no conflict of interest.

## Author contributions

BL and LT wrote the manuscript with the input of all authors. LT, RH, WX, RH, SP, and SP carried out the experiments and performed the analyses. LT, BL, and UW supervised the project. BL and LT conceived the original idea, contributed to the design and implementation of the research. LT, RH, and WX prepared the figures.

## Supporting information


**Fig. S1.** Series of constructs used in the present study
**Fig. S2.** Map of pcDNA3.1‐FLEX.
**Fig. S3.** Map of pLV‐hsyn‐GFP‐WPRE.
**Fig. S4.** Map of pLV‐hsyn‐FLEX‐rev‐GFP.
**Fig. S5.** LV‐hSyn‐GFP‐WPRE targets GFP expression to neurons.
**Table S1.** Sequences of oligonucleotides.

## Data Availability

All raw data are available upon request to the corresponding author.
