# Supplementary material for: A Cre‐dependent lentiviral vector for neuron subtype‐specific expression of large proteins
Source: FEBS Lett. 2025 Oct 28;600(3):342–55. doi: 10.1002/1873-3468.70205 (PMC12883904; doi:10.1002/1873-3468.70205)
Supplement: Supplementary file 1 — Fig. S1. Series of constructs used in the present study Fig. S2. Map of pcDNA3.1‐FLEX. Fig. S3. Map of pLV‐hsyn‐GFP‐WPRE. Fig. S4. Map of pLV‐hsyn‐FLEX‐rev‐GFP. Fig. S5. LV‐hSyn‐GFP‐WPRE targets GFP expression to neurons. Table S1. Sequences of oligonucleotides. [file FEB2-600-342-s001.docx]

**A Cre-dependent lentiviral vector for neuron subtype-specific expression of large proteins - *Xu et al.***

**Supplementary material**

Figure S1 1

Figure S2 2

Figure S3 3

Figure S4 4

Figure S5 5

Table S1 6


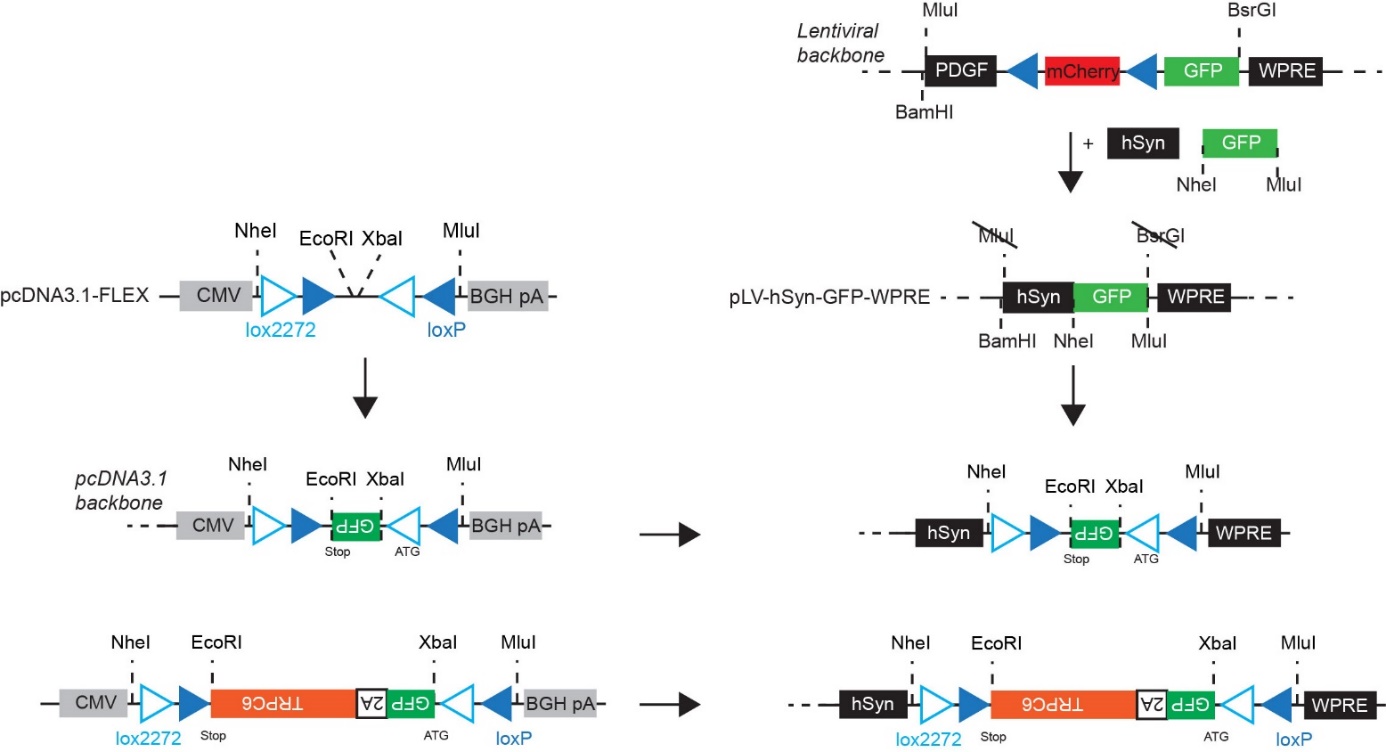


**Figure S1: Series of constructs used in the present study**

The plasmid pcDNA3.1-FLEX, which comprises a FLEX switch was custom made and used to assemble Cre-dependent expression cassettes before transfer into the lentivector.

pLV-hSyn-GFP-WPRE was derived from pLV-PDGF-lox-mCherry-lox-EGFP and was used as acceptor of Cre-dependent expression cassettes.


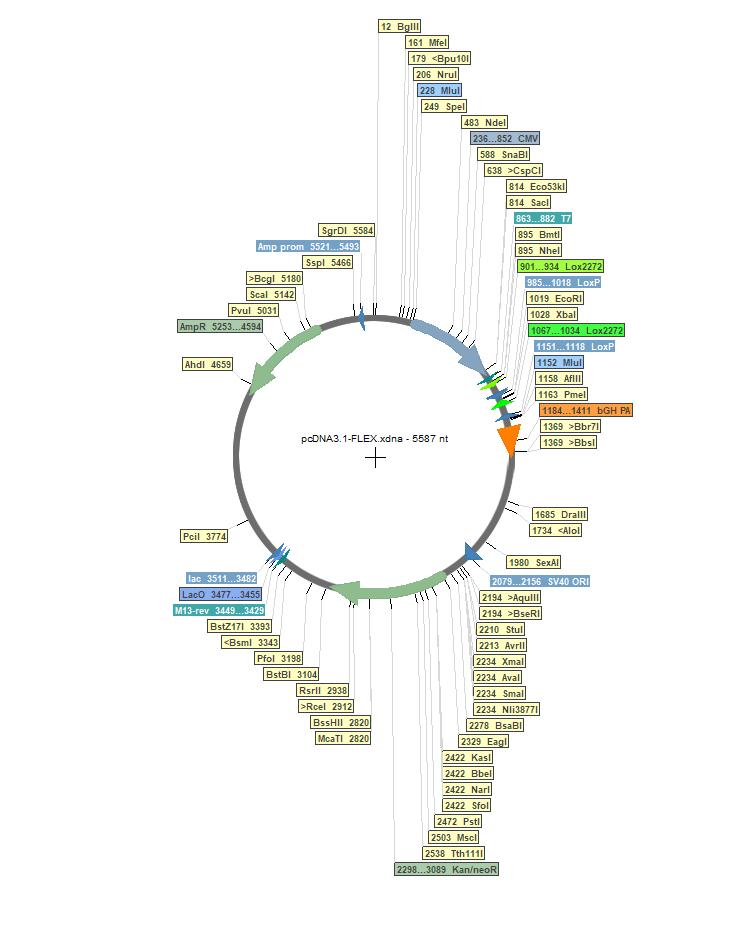
 **Figure S2: Map of pcDNA3.1-FLEX**


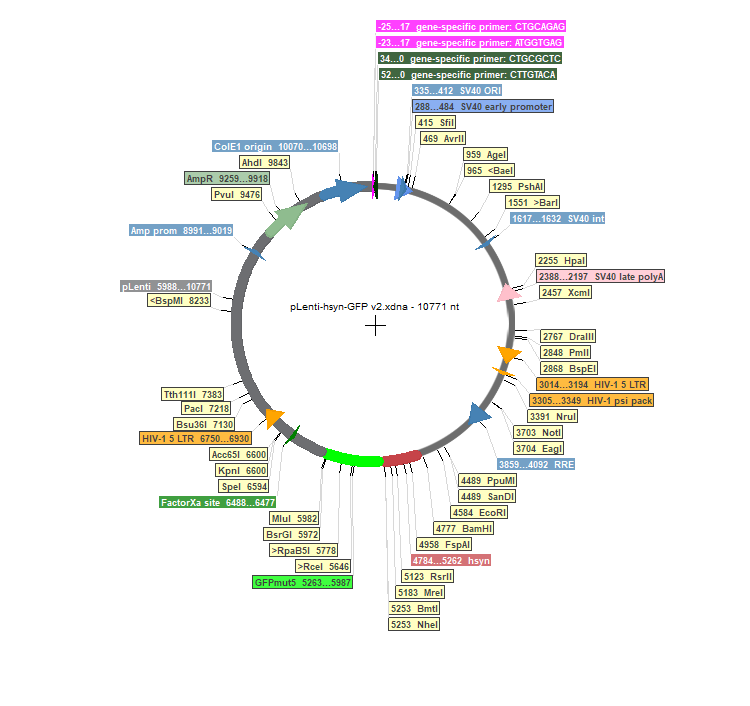
 **Figure S3 : Map of pLV-hsyn-GFP-WPRE**


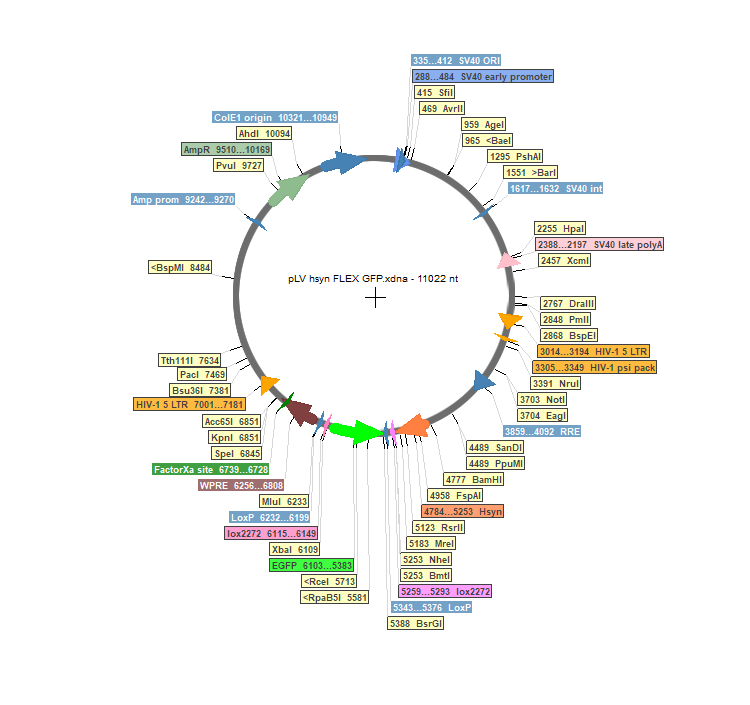
 **Figure S4: Map of pLV-hsyn-FLEX-rev-GFP**


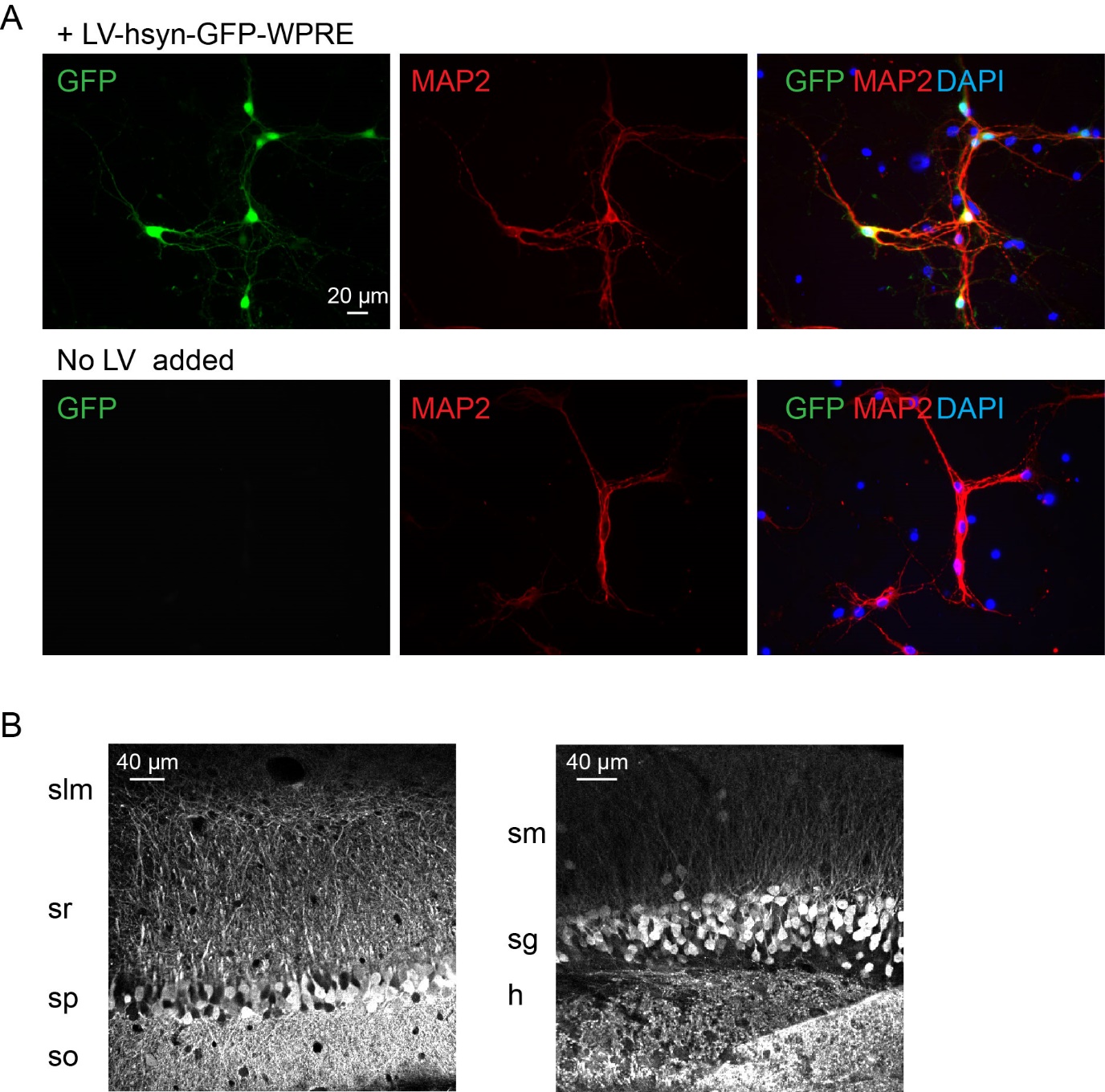


**Figure S5: LV-hSyn-GFP-WPRE targets GFP expression to neurons**. A. Fluorescence image of primary cortical neurons transduced by LV-hSyn-GFP-WPRE and immunostained against GFP (green) and the neuronal marker MAP2 (red). Nuclei are counterstained with DAPI (blue). B. Expression of GFP in CA1 (left) and dentate gyrus (right) of hippocampus one month after transduction with LV-hSyn-GFP-WPRE. so:stratum oriens, sp: stratum pyramidale, sr:stratum radiatum, slm: stratum lacunosum moleculare, h: hilus, sg: stratum granulare, sm: stratum moleculare.

**Table S1 : Sequences of oligonucleotides**

| Name | Sequence | Amplify |
| --- | --- | --- |
|  | *For pLV-hsyn-GFP-WPRE* |  |
| Lenti-hsyn-For | ccactttggctgatacgcggatccaCTGCAGAGGGCCCTGCGT | hsyn promoter |
| GFP-hsyn-Rev | caccatggtggctagcCTGCGCTCTCAGGCACGAC | hsyn promoter |
| Hsyn-GFP-For | cctgagagcgcaggctagccaccATGGTGAGCAAGGGCGAG | GFP |
| WPRE-GFP-Rev | gttgattatcgaggccgctttacttacgcgtcaCTTGTACAGCTCGTCCATGC | GFP |
|  |  |  |
|  | *For pcDNA3.1-FLEX-rev-GFP* |  |
| FLEX | GCTAGCAtaacttcgtataggatactttatacgaagttatgcagaatggtagctggattgtagctgctattagcaatatgaaacctcttaataacttcgtatagcatacattatacgaagttatGAATTCatgTCTAGA  ataacttcgtataaagtatcctatacgaagttatttgccttaacccagaaattatcactgttattctttagaatggtgcaaagaataacttcgtataatgtatgctatacgaagttatACGCGT |  |
| FLEX-GFP-For | gacTCTAGagccaccATGGTGAGCAAGGGC | GFP |
| FLEX-GFP-Rev | cagGAATTCTTACTTGTACAGCTCGTCCAT | GFP |
|  |  |  |
|  | *For pcDNA3.1-GFP-2A-TRPC6DN* |  |
| pcDNA-GFP-For | ctagcgtttaaacgggccctctagaGCCACCATGGTGAGCAAG | GFP |
| 2A-GFP-Rev | tagtagctccgcttccCTTGTACAGCTCGTCCATG | GFP |
| 2A-TRPC6-For | gctgtacaagggaagcggagctactaacttcagcctgctgaagcaggctggagacgtggaggagaaccctggacctAGCCAGAGCCCGAGGTTC | TRPC6 |
| pcDNA-TRPC6-Rev | ccactagtccagtgtggtggaattCTATCTGCGGCTTTCCTCCAG | TRPC6 |

NheI, Lox2272, loxP, EcoRI, XbaI, MluI, BamHI
